# AMPK regulates ARF1 localization to membrane contact sites to facilitate fatty acid transfer between lipid droplets and mitochondria

**DOI:** 10.1038/s41419-025-07957-7

**Published:** 2025-08-18

**Authors:** Lupeng Chen, Yue Liu, Junzhi Zhang, Tongxing Song, Jian Wu, Zhuqing Ren

**Affiliations:** 1 Key Laboratory of Agriculture Animal Genetics, Breeding and Reproduction of the Ministry of Education, College of Animal Science and Technology, Huazhong Agricultural University, Wuhan, Hubei 430070 China; 2 Frontiers Science Center for Animal Breeding and Sustainable Production, Wuhan, Hubei 430070 China; 3 Hubei Hongshan Laboratory, Wuhan, Hubei 430070 China

**Keywords:** Energy metabolism, Organelles

## Abstract

Lipid droplet (LD) -mitochondrion contacts play a crucial role in regulating energy metabolism and fatty acid oxidation in skeletal muscle cells. However, the proteins that regulate these interactions remain poorly understood. Here, we demonstrate that the binding between ADP-ribosylation factor 1(ARF1) and perilipin2 (Plin2) regulates LD-mitochondrion contacts under starvation conditions, facilitating the transfer of fatty acids from LDs to mitochondria. In C2C12 cells, starvation increased ARF1’s GTP-binding activity and its localization to mitochondria, enhancing ARF1’s binding to Plin2 and facilitating fatty acid flow from LDs to mitochondria. In contrast, knockdown of ARF1 reduced LD-mitochondrion interactions and blocked fatty acids transfer. Additionally, ARF1-mediated interactions were regulated by AMPK; inhibiting AMPK activity reduced ARF1 localization to LDs and mitochondria, and blocked LD-mitochondrion interactions. In mice, starvation increased ARF1 expression in muscle tissue and LD-mitochondrion contacts. Conversely, inhibiting ARF1 led to lipid accumulation in muscle tissue. In conclusion, our work suggests that ARF1 is a critical regulator of LD-mitochondrion interactions and plays a significant role in energy metabolism regulation in skeletal muscle.

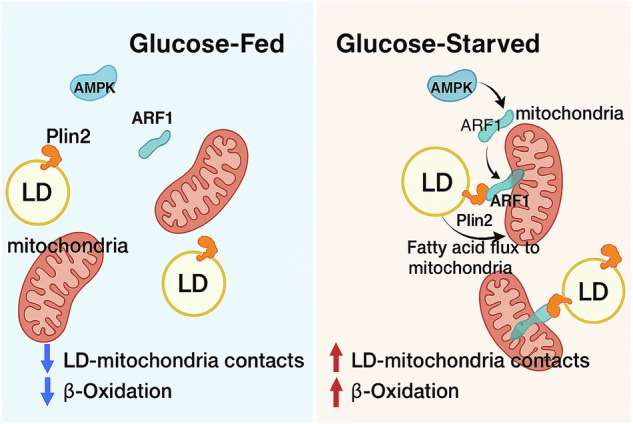

## Introduction

Skeletal muscle plays a crucial role in maintaining the body’s energy balance, accounting for approximately 40% of total body mass [1]. It is a primary site for glucose and fatty acid oxidation and can adapt to fluctuations in nutrient availability to support cellular function [2]. During periods of nutrient scarcity, such as starvation, skeletal muscle shifts from relying on glucose to utilizing fatty acids stored in lipid droplets (LDs) as an internal energy source [3]. This metabolic flexibility is vital for sustaining cellular energy production, enabling muscles to meet increased ATP demands without depending on external nutrients [3].

LDs in skeletal muscle play a vital role in storing and mobilizing lipids, particularly during metabolic stress [4]. In response to starvation, LDs undergo remodeling, increasing their interaction with mitochondria to facilitate the release of fatty acids, which are then transported into mitochondria for β-oxidation [5]. This LD-mitochondrion contact is crucial for cellular energy production and involves a complex network of proteins that regulate LD dynamics, mitochondrial function, and fatty acid transfer [6–8]. Plin5 has been identified as a key regulator of LD-mitochondrial contact in oxidative tissues [9], including the heart, liver, muscle, and brown adipose tissue. In skeletal muscle, this contact is mediated by a tethered chain structure composed of Plin5 and either Rab8a [10] or FATP4 [11]. Recently, studies in cardiac muscle have also highlighted the roles of MFN2 and Hsc70 in mediating LD-mitochondrion contact, promoting fatty acid oxidation [12]. However, the presence of a Plin5-independent regulator of LD-mitochondrion interactions in skeletal muscle remains uncertain.

Recent studies have revealed that small GTPases, traditionally known for their roles in vesicular trafficking, are also integral components of the lipid droplet (LD) proteome. Enzymes such as RAB5, RAB7, RAB8, RAB18, and ADP-ribosylation factor 1 (ARF1) have been implicated in regulating contacts between LDs and other organelles [10, 13–16]. For example, GDP-bound Rab8A localizes to LD contact sites and facilitates lipid transfer from smaller to larger LDs via Fsp27 [14], whereas its GTP-bound form interacts with Plin5 to mediate LD–mitochondria contacts [10]. Similarly, ARL8B forms heterotypic complexes that target LD–lysosome contact sites, promoting efficient lipid exchange between these compartments [17].

ARF1, in particular, has emerged as a critical regulator of lipid metabolism [18]. While its established roles in Golgi function and endosomal trafficking are well documented [19], its specific mechanisms in modulating skeletal muscle energy homeostasis remain poorly understood. Recent evidence indicates that ARF1 not only promotes the formation of contacts between LDs and the endoplasmic reticulum [16] but also plays a key role in regulating LD dynamics and facilitating interactions between LDs and mitochondria [20]. However, the precise molecular functions of ARF1 in these processes are not yet fully elucidated. As such, ARF1 may serve as a crucial node in coordinating the mobilization of stored lipids with mitochondrial energy production, a hypothesis that warrants further investigation.

ADP-ribosylation factor 1 (ARF1), a small GTPase traditionally linked to vesicular transport and membrane dynamics, is emerging as a key regulator of lipid metabolism. [18]. While ARF1’s role in cellular processes such as Golgi function and endosomal transport is well established [19], its involvement in regulating energy homeostasis in skeletal muscle remains less clear. Recent evidence suggests that ARF1 plays a critical role in regulating LD dynamics and facilitating interactions between LDs and mitochondria [20]. However, its precise molecular function in this process is still not fully understood. ARF1 may serve as a crucial link in coordinating the mobilization of storage lipids with mitochondrial energy production, but the signaling pathways and regulatory mechanisms underlying this process are yet to be fully elucidated.

In the present study, we found that glucose starvation promotes LD-mitochondrion contact and increases ARF1 expression. Overexpression of ARF1 enhances LD-mitochondrion contact and facilitates fatty acid transfer between these two organelles, whereas knockdown of ARF1 blocks this process. Further investigations revealed that ARF1 localizes at the contact site between LDs and mitochondria. Mechanistically, ARF1’s regulation of LD-mitochondrion interactions depends on its active form and its binding to Plin2. ARF1-Q71L showed the strongest binding capacity to Plin2, whereas ARF1-T31N had a much weaker binding capacity. Additionally, ARF1-mediated interactions between LDs and mitochondria are regulated by AMPK signaling, with AMPK activation inducing the localization of active ARF1 in mitochondria. In vivo, starvation induces ARF1 expression and LD-mitochondrion contact in mouse muscle tissue, while administration of an ARF1 inhibitor blocks this process. In conclusion, our results suggest that ARF1 mediates the interaction between LDs and mitochondria and regulates fatty acid transfer.

## Methods

### Mice

The C57BL/6 J mice were purchased from the Laboratory Animal Center of Three Gorges University. All mice were housed in a normal environment and provided with food and water. All experimental protocols were approved by the Ethics Committee of Huazhong Agricultural University. The methods were carried out in accordance with the approved guidelines from Huazhong Agricultural University and the scientific, ethical, and legal principles of the Hubei Regulations for the Administration of Affairs Concerning Experimental Animals. All of the experimental protocols were subject to approval by the Ethics Committee of Huazhong Agricultural University (HZAUMU2013-0005).

### Inhibitor BFA injection

The ARF1 inhibitor Brefeldin A (BFA), purchased from MedChemExpress (catalog number HY-16592), was first dissolved in DMSO to create a 20 μg stock solution. This stock solution was not directly used for injection into the muscle tissues of C57BL/6 mice. Instead, it was further diluted in a 1:9 ratio with a saline solution containing 20% Sulfobutylether-β-Cyclodextrin (SBE-β-CD), thoroughly mixed to obtain the working solution. The working solution was administered at a dose of 4.0 mg/kg [21] into the medial thigh muscle of the right hindlimb, while the left hindlimb received an equivalent volume of a DMSO and 20% SBE-β-CD saline mixture as the control. The injections were administered on days 1, 3, and 5, with 18 7-week-old mice of similar physiological status randomly divided into three groups, each consisting of 6 mice.

### Cell culture and transfection

C2C12 cells were purchased from the National Collection of Authenticated Cell Cultures and cultured in Dulbecco’s Modified Eagle’s Medium (DMEM) (HyClone), supplemented with 10% fetal bovine serum (AusGeneX, Molendina, Australia), 100 units/mL of penicillin, and 100 μg/mL of streptomycin (HyClone). The cells were cultured in Petri dishes at 37 °C and transfected using Lipo3000™ Transfection Reagent (Thermo Fisher). For transfection, C2C12 cells were seeded onto cell slides in 6-well plates and transfected with plasmid vectors following the manufacturer’s instructions for the transfection reagent.

To prepare the oleic acid treatment solution, a PBS solution containing 20 mM oleic acid was first prepared and heated in a water bath at 70 °C for 30 min until fully dissolved. This solution was then combined with culture medium supplemented with 20% fatty acid (FA)-free bovine serum albumin (BSA) in a 37 °C water bath. Prior to treatment, cells cultured on slides or plates were rinsed three times with PBS, after which the appropriate volume of the oleic acid working solution was added to the wells. The cells were then incubated for 24 h.

Following the oleic acid treatment, for glucose starvation, the medium was replaced with a low-glucose medium (#11885076) containing 1000 mg/L glucose, and the cells were incubated for 8 h. In contrast, the control group was treated with medium containing normal glucose concentration (#11965126, Thermo Fisher), which has a glucose concentration of 4500 mg/L.

### Fluorescent FA tracking assay

C2C12 cells were first labeled with 1 μM Bodipy 558/568 C12 (red C12, D-3835, Thermo Fisher Scientific) in DMEM medium containing 10% fetal bovine serum (FBS) for 16 h. Afterward, the cells were washed three times with DPBS to remove excess red C12. The cells were then co-incubated with serum-enriched medium for 1 h to allow fluorescent lipids to enter the LDs. Following this, the cells were incubated in either high-glucose or low-glucose medium for 8 h. To label the mitochondria and LDs, Mito-Tracker Red CMXRos (C1035, Beyotime) and Bodipy 493/503 (D3922, Thermo Fisher Scientific) were used, respectively. Imaging was performed using the AX R with NSPARC (Nikon Spatial Array Confocal) microscope for visualization of both mitochondria and LDs.

### Plasmid DNA construction

Full-length coding sequences encoding ARF1 (NM_001130408.2) was amplified using a cDNA library of C2C12 cells and then subcloned into the pcDNA3.1 vector (Beyotime) and pEGFP-C1 and pmCherry-C1 vectors (Huayu Gene). The fluorescent labeling vectors pCMV-mCherry and pCMV-C-EGFP were purchased from Wuhan Tianyi Huayu Gene Technology. The gene expression vectors pCMV-N-Flag and pCMV-N-HA were purchased from Nanjing Biyuntian Biotechnology.

### Hematoxylin-Eosin Staining and Oil Red O Staining of Histological Sections

For HE staining, the Skeletal muscle tissues were divided and fixed in 4% paraformaldehyde for 24 h. After dehydration, the tissues were embedded in paraffin for sectioning. After the dewaxing process, the sections were stained with hematoxylin for 8–10 min. Sections were dehydrated again and were stained with eosin for 3 min. After dehydration, the sections were sealed with neutral gum. For Oil Red O staining, the liver tissues were fixed in 10% formalin and were prepared for frozen sections at a thickness of 6–10 μm. The sections were mounted on slides and were dried for 10 min at room temperature. The sections were incubated in 75% alcohol for 10 s and were stained in Oil Red O solution (#G1016, Servicebio). The slides were differentiated for 1 min until the background was colorless. After washing for 2 min, the sections were stained with hematoxylin for 8 min. The sections were washed and differentiated in a 1% aqueous HCl solution for 1 min. The sections were incubated in ammonia for several minutes. The slides were sealed with a glycerin gelatin solution.

### LDs marking and observation

Cells were seeded on slides in a 24-well plate and were cultured for 24 h. The slides were fixed in 4% paraformaldehyde for 15 min at room temperature. The slides were stained with BODIPY 493/503 (#D3922, Invitrogen, Carlsbad, CA, USA) for 45 min at 37 °C and were then stained with DAPI (#G-1012, Servicebio) for 10 min at 37 °C. After washing three times with PBS for 10 min each, the slides were sealed with an anti-fluorescent quenching solution for microscopic observation.

### Western Blot and Real-Time PCR

Real-time PCR was performed using the QuantStudio 6 Flex Real-Time PCR System (ABI, Thermo Fisher, Shanghai, China) and the following PCR program: denaturation at 95 °C for 2 min; amplification for 40 cycles at 95 °C for 10 s; annealing and extension at 60 °C for 32 s. Primer sequences are shown in Table 1. Specific amplification for certain PCR reactions was assessed using a melting curve. One negative control reaction, in which the cDNA template was replaced by water, was performed to avoid potential contamination. The sample from each well was repeated three times, and the comparative Ct (ΔΔCt) value method was used for relative quantification. GAPDH (NM_002046.6) was used as the reference gene.

**Table 1:** Quantitative RCR primer sequences.

gene name	Forward (5’-3’)	Reverse (5’-3’)
ARF1	CAAGCAGGACCTCCCCAATG	CGGAGCTGATTAGACAGCCA
PLIN1	CAAGCACCTCTGACAAGGTTC	GTTGGCGGCATATTCTGCTG
PLIN2	TCTGCGGCCATGACAAGTG	GCAGGCATAGGTATTGGCAAC
PLIN3	GTGTGGGACAGATGGTGATTAG	GCCCAACCGGACAAAGTAGT
PLIN4	GTGTCCACCAACTCACAGATG	GGACCATTCCTTTTGCAGCAT
PLIN5	CTTCCTGCCCATGACTGAGG	GACCCCAGACGCACAAAGTAG
DGAT1	GTGCCATCGTCTGCAAGATTC	GCATCACCACACACCAATTCAG
DGAT2	GCGCTACTTCCGAGACTACTT	GGGCCTTATGCCAGGAAACT
ACSL3	TGTCTTTCTCATGGATGCCGA	CAGCACGGATGTGTCTCCTT
SREBF1	CTTTGGCCTCGCTTTTCGG	TGGGTCCAATTAGAGCCATCTC
FASN	AGAGATCCCGAGACGCTTCT	GCTTGGTCCTTTGAAGTCGAAGA
SCD1	TTCTTGCGATACACTCTGGTGC	CGGGATTGAATGTTCTTGTCGT
PPARG	GGAAGACCACTCGCATTCCTT	GTAATCAGCAACCATTGGGTCA
FSP27	ATGGACTACGCCATGAAGTCT	CGGTGCTAACACGACAGGG
FITM1	CCTCTGCCTTACTGTACTTTGG	TAGCGAAGATCGTCCGAGAGT
FITM2	TCATTGCCCTTACCAACTACCA	GAGTGGCCCGAGATGTCAAA
BSCL2	CTGTTGCCAATGTCTCACTGG	TCTAAGGTGACTCGATATGGCTG
FABP4	AAGGTGAAGAGCATCATAACCCT	TCACGCCTTTCATAACACATTCC
HSL	GATTTACGCACGATGACACAGT	ACCTGCAAAGACATTAGACAGC
ATGL	ATGTTCCCGAGGGAGACCAA	GAGGCTCCGTAGATGTGAGTG
MGL	AGGCGAACTCCACAGAATGTT	ACAAAAGAGGTACTGTCCGTCT
NDUFV1	GTGCGGGTATCTGTGCGTT	GGTTGGTAAAGATCCGGTCTTC
SDHB	ATTTACCGATGGGACCCAGAC	GTCCGCACTTATTCAGATCCAC
COX7C	ATGTTGGGCCAGAGTATCCG	ACCCAGATCCAAAGTACACGG
CS	GGACAATTTTCCAACCAATCTGC	AGTCAATGGCTCCGATACTGC
ATP5F1	AGTTCCTTTACCCTAAGACTGGT	TTCATGCTCGACTGCTTTACTT
GAPDH	AGGTCGGTGTGAACGGATTTG	GGGGTCGTTGATGGCAACA

For protein extraction, cells were plated in six-well plates with approximately 1×10^6 cells per well. Transfected cells were homogenized in 1 mL of 25 mM Tris/1 mM ethylenediaminetetraacetic acid pH buffer (pH 7.5). Homogenates were separated by 12.5% sodium dodecyl sulfate-polyacrylamide gel electrophoresis (SDS-PAGE) and were transferred to a polyvinylidene fluoride (PVDF) membrane (Millipore, Bedford, MA, USA) using a semidry electrophoretic method. The blocked membranes (5% BSA in tris-buffered saline (TBS) buffer containing 0.1% Tween 20) were incubated with antibodies overnight at 4 °C. The blots were extensively washed three times with tris-buffered saline with Tween 20 (TBST) buffer for 10 min and were incubated under gentle agitation with the secondary antibodies for immunodetection. The antigen–antibody reaction was incubated for 1 h, and the cross-reacting proteins were detected with ECL chemiluminescent detection reagent (Shandong Sparkjade Biotechnology Co.， Ltd., #ED0015-C). Pre-stained molecular weight markers 10–170 kD in weight (Fermentas, Burlington, ON, Canada) were used as standards.

### Immunofluorescence assay

For immunofluorescence imaging, cells were seeded into 24-well plates or confocal petri dishes, with approximately 3 × 10^5 cells per well in the 24-well plates and 5 × 10^5 cells in the confocal petri dishes. Cells on the slides were fixed in 4% paraformaldehyde for 15 min at room temperature. The paraformaldehyde was removed, and the cells were washed with PBS three times for 10 min each. The slides were permeabilized using 0.5% Triton X-100 for 40 min at 37 °C. Next, the 0.5% Triton X-100 was removed and the cells were again washed with PBS three times for 10 min each. Using a 3% BSA solution, the slides were blocked for two hours at 37 °C. The blocking solution was removed, and the primary antibody was diluted in the first solution (#NKB-301, Can Get Signal Immunoreaction Enhancer Solution, TOYOBO, Shanghai, China) was added to the cell wells for a 16 h incubation at 4 °C. The primary antibody solution was removed, and the slides were washed with PBS three times for 10 min each. The second antibody diluted in a second solution (#NKB-301, Can Get Signal Immunoreaction Enhancer Solution, TOYOBO) was added to the cell wells and was incubated for 1 h at 37 °C. The slides were washed with PBS three times for 10 min each. The slides were stained with BODIPY 493/503 (#D3922, Invitrogen) for 45 min at 37 °C and were then stained with DAPI (#G-1012, Servicebio) for 10 min at 37 °C. After washing with PBS three times for 10 min each, the slides were sealed with an anti-fluorescent quenching solution. The confocal laser scanning microscope (Carle Zeiss, German) was used to observe the slide of cells.

### Image analysis

Confocal images were analyzed using ImageJ for quantification. Quantification of LD number and area usingimageJ: First, the contrast of the fluorescent image of LDs was adjusted to enhance visibility. The image was then thresholded to separate the LDs from the background, with the threshold level manually adjusted to ensure clear delineation of the droplets. The resulting binary image was processed to reduce noise by removing outliers, followed by morphology filtering. To quantify the droplets, particle analysis was performed, with appropriate size filters applied to exclude background noise. The particle counts, areas, and other morphological features were recorded in the results window. Statistical data were then exported and plotted using GraphPad Prism.

To assess the colocalization of LDs and mitochondria, a particle-based approach was employed. First, the images were converted to binary format, and the colocalized points between LDs and mitochondria were quantified using the “Co-localization” plugin in ImageJ. Next, the “Analyze Particles” function was used to quantify the number of double-colocalized pixels. These colocalized particles were then expressed as a percentage of the total number of LDs, representing the proportion of LDs in contact with mitochondria relative to the total number of LDs in the cell. To assess the connectivity between mitochondria and LDs, a mask was generated from the segmented mitochondrial images. The percentage of mitochondrial signal in proximity to LDs, relative to the total mitochondrial signal, was then calculated.

To analyze LD-related ARF1, the images were imported into ImageJ, and individual LDs were detected using the “Analyze Particles” function. Larger regions of interest (ROIs) around each LD were generated with the “Dilate” function to include the surrounding signal of interest. The fluorescent signal intensities associated with the LDs were measured, normalized, and plotted, and similar analysis was performed for mitochondria associated ARF1.

The colocalization of Bodipy 558/568 C12 with LDs and mitochondria was quantified using Manders’ overlap coefficient, calculated with ImageJ software.

In analyzing the fluorescence signal intensity of ARF1 at the contact site between LDs and mitochondria, we utilized the ROI Manager function in ImageJ software. This allowed us to precisely locate the same positions across different color channels. Fluorescence intensity was then measured for each channel at these specific points, and the resulting data were plotted using GraphPad Prism for visualization.

### Antibodies For IF and WB

IF: 1:100, WB: 1:2000, Rabbit polyclonal anti-ARF1, Proteintech, Cat #10790-1-AP

WB: 1:2000, Rabbit polyclonal anti-AMPK, Proteintech, Cat #10929-2-AP

WB: 1:2000, Mouse monoclonal anti-p-AMPK, Proteintech, Cat #CL488-66536

WB: 1:2000, Rabbit polyclonal anti-ACTIN, Proteintech, Cat #23660-1-AP

WB: 1:2000, Rabbit polyclonal anti-GAPDH, Proteintech, Cat #10494-1-AP

WB: 1:2000, Rabbit polyclonal anti-ADRP, Proteintech, Cat #15294-1-AP

WB: 1:2000, Rabbit polyclonal anti-ATGL, Abclonal, Cat #A6245

WB: 1:2000, Rabbit polyclonal anti-HSL, Abclonal, Cat #A15686

WB: 1:2000, Rabbit GFP tag Polyclonal antibody, Proteintech, Cat #50430-2-AP

WB: 1:2000, ChromoTek mouse anti RFP Monoclonal antibody, Proteintech, Cat #6g6

WB: 1:2000, Rabbit polyclonal anti-TOM20, Abclonal, Cat #A21723

WB: 1:8000, HRP Goat Anti-Rabbit IgG (H + L), Affinity Biosciences, Cat #S0001

WB: 1:8000, HRP Goat Anti-Mouse IgG (H + L), Affinity Biosciences, Cat#S0002

IF: 1:200, Cy3 Goat Anti-Rabbit IgG (H + L), Abclonal, Cat #AS007

IF: 1:200, Cy3 Goat Anti-Mouse IgG (H + L), Abclonal, Cat #AS008

IF: 1:200, FITC Goat Anti-Rabbit IgG (H + L), Abclonal, Cat #AS011

IF: 1:200, FITC Goat Anti-Mouse IgG (H + L), Abclonal, Cat #AS001

### Triglyceride content detection

The TG content of the liver tissues was detected by fully automatic chemistry analyser (#Chemray 240, Rayto Life and Analytical Sciences Co.,Ltd., Shenzhen, China). The total protein concentration was detected by Enzyme Label Detector (#Epoch, BioTek Instruments, Inc., headquartered in Winooski, VT, USA). The TG content data was normalized by protein concentrations, mmol/g.

### LD isolation

For skeletal muscle LDs isolation, the tissue was first cut into small fragments using spring scissors and transferred to an electric homogenizer containing HES buffer (20 mM HEPES, 1 mM EDTA, 250 mM sucrose). The homogenate was then filtered through double gauze and centrifuged at 2000 g for 5 min. The lower clear liquid was discarded, and the LDs floating in the upper layer were carefully transferred to a 5 mL tube using a wide-mouth pipette, followed by two washes with HES buffer. The LDs were then transferred to Ultra-Clear ultracentrifuge tubes (Beckman-Coulter), adjusted to a final sucrose concentration of 20%, and layered with 5% sucrose/HE buffer and HES buffer. The tubes were centrifuged at 16,0000 g for 30 min at 4 °C, and the buoyant LDs were collected.

C2C12 cells were cultured to 90% confluence in 15 cm dishes and treated with 400 µM oleic acid in growth medium for 24 h. After treatment, the cells were incubated for 8 h with normal DMEM containing 10% fetal bovine serum. The glucose-fed group was incubated with normal DMEM containing 10% fetal bovine serum, while the glucose-starved group was incubated with low-glucose DMEM containing 10% fetal bovine serum for 8 h. After scraping, the cells were collected in 2 mL of lysis buffer (25 mM Tris-HCl, pH 7.4, 100 mM KCl, 1 mM EDTA, 5 mM EGTA, and a protease inhibitor cocktail) and lysed through a 27-gauge needle. The lysate was centrifuged at 1,500 g for 5 minutes, and the supernatant was adjusted to a final volume of 2.5 mL with 20% sucrose. This supernatant was transferred to a 10 mL polycarbonate ultracentrifuge tube, and sequential layers of 2.5 mL of 10% sucrose, 2.5 mL of 4.2% sucrose, and 2.5 mL of lysis buffer were added. The tubes were then centrifuged at 150,000 g for 1 hour at 4 °C, and the buoyant LDs were collected.

### Mitochondrial isolation

Mitochondrial isolation was performed using the Beyotime Biotechnology kit (C3601). First, cells were collected and washed with ice-cold PBS, then resuspended in mitochondrial isolation reagent containing PMSF and incubated on ice for 10–15 min. The cell suspension was then transferred to a glass homogenizer and homogenized. The effectiveness of homogenization was assessed using Trypan Blue staining, ensuring that more than 50% of the cells were blue. After homogenization, the cells were centrifuged at 600 g for 10 min at 4 °C to remove cellular debris. The supernatant was transferred to a new tube and centrifuged at 11,000 g for 10 minutes at 4 °C to precipitate the mitochondria.

### Bioinformatics and data analysis

The protein-protein interaction network was analyzed using STRING (https://string-db.org/). Briefly, LD-associated proteins were co-entered with ARF1 into the “Multiple Proteins” module, and the “SEARCH” function was used to analyze the interactions.

### Statistical analyses

All of the experiments were repeated three times. Data were extracted as the mean ± SD. We used the unpaired Student’s t-test to compare differences between two independent sample groups. For comparisons involving more than two groups, we applied one-way or two-way analysis of variance (ANOVA), followed by Tukey’s post hoc multiple comparison test.

### Statements of approval

We confirm that all methods were performed in accordance with the relevant guidelines and regulations of Ethics Committee of Huazhong Agricultural University.

## Results

### Glucose starvation treatment promotes LD-mitochondrion contact and ARF1 expression

**Fig. 1:**
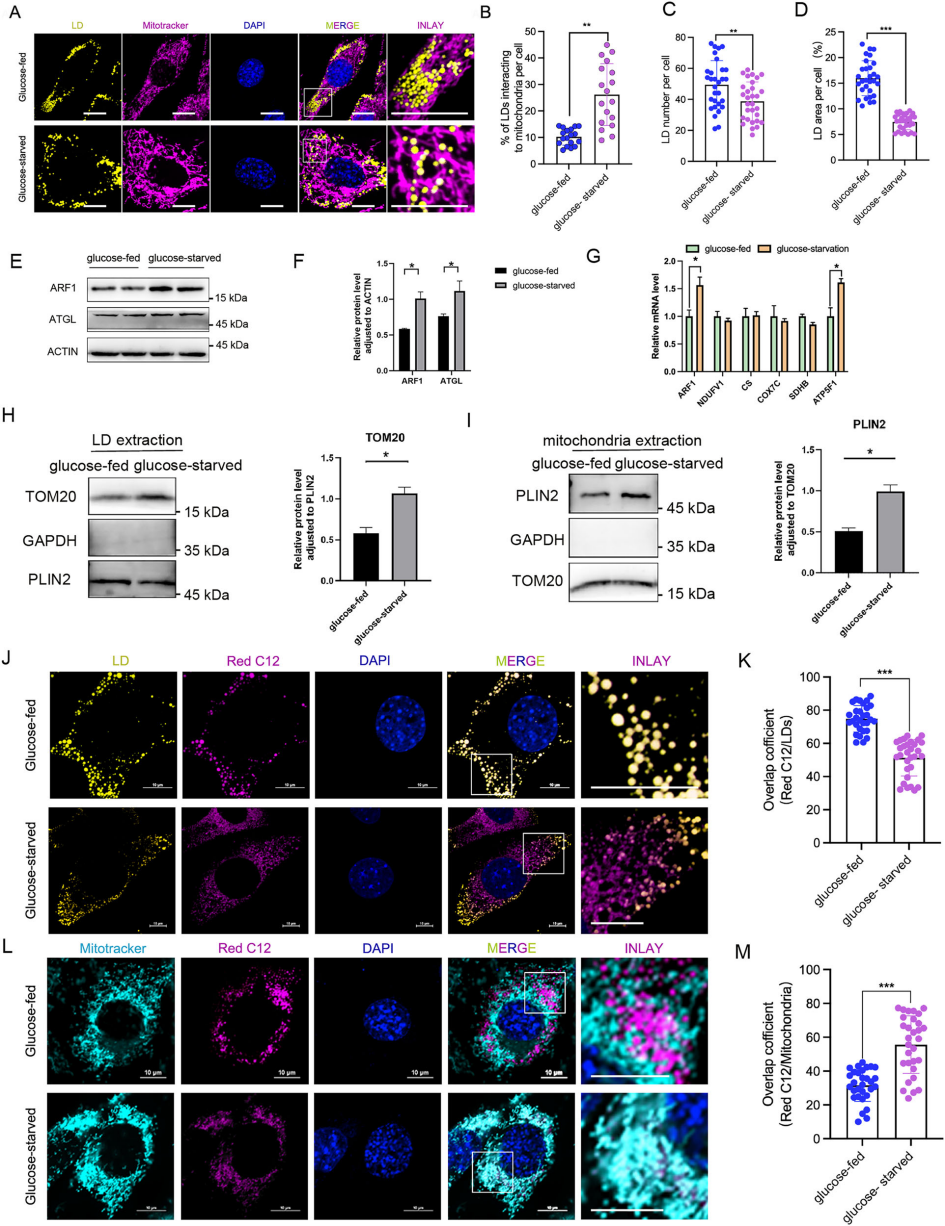
Glucose starvation promotes LD-mitochondrion contact and LD-mitochondrion fatty acid transfer as well as ARF1 expression. **A**, **B** Representative optical section images and quantification results of LD-Mito contacts in C2C12 cells treated with glucose starvation for 8 hours using MitoTracker (mitochondrial marker, red) and BODIPY493/503 (LD marker, green) staining. *n* = 18 cells for colocalization quantification using ImageJ software. Scale bars, 10 μm. **C**, **D** Quantification of LD number and area per cell. *n* = 30 cells. **E** Western blot (WB) analysis for ARF1 and ATGL in C2C12 cells from glucose-starved (*n* = 3) and glucose-fed (*n* = 3) groups. **F** Quantification of WB results for ARF1 and ATGL normalized to ACTIN from (**E**). **G** Relative mRNA expression of ARF1 and β-oxidation-related genes detected by qPCR in C2C12 cells from glucose-starved (*n* = 3) and glucose-fed (*n* = 3) groups. **H** WB analysis of TOM20, Plin2, and GAPDH in LDs isolated from C2C12 cells from glucose-starved (*n* = 3) and glucose-fed (*n* = 3) groups. Quantification of TOM20 normalized to Plin2 (right panel). **I** WB analysis of TOM20, Plin2, and GAPDH in mitochondria isolated from glucose-starved (*n* = 3) and glucose-fed (*n* = 3) C2C12 cells. Quantification of Plin2 normalized to TOM20 (right panel). **J**–**M** LCFA tracking assay in C2C12 cells from glucose-starved and glucose-fed groups. **J** C2C12 cells were incubated with Red C12 for 16 h and then treated with or without glucose deprivation for 8 h before staining with BODIPY493/503 (LD marker, green). Representative optical section images are shown. **K** Quantification of Red C12/LD overlap coefficient. *n* = 24 cells. **L** Representative optical section images of C2C12 cells incubated with Red C12 for 16 h and then treated with or without glucose deprivation for 8 h before staining with MitoTracker (mitochondrial marker, green). **M** Quantification of Red C12/mitochondrion overlap coefficient. *n* = 24 cells. **B**–**D**, **F**, **H**, **I**, **K**, **M** Data are presented as means ± SD of three biologically independent replicates, analyzed by two-sided unpaired Student’s t-test.*p < 0.05, **p < 0.01, ***p < 0.001.

To investigate the dynamic changes in LD-mitochondrion interactions under starvation conditions, we subjected C2C12 cells to three different starvation treatments: glucose deprivation, serum deprivation, and simultaneous deprivation of both serum and glucose. We used immunofluorescence to detect the contact between LDs and mitochondria. We found that all three types of starvation significantly promoted the contact between LDs and mitochondria compared to the control (Fig. 1A, B and Fig. S1A-D). Additionally, glucose starvation significantly reduced the number and area of LDs (Fig. 1C, D), suggesting that glucose deprivation promotes LD depletion in C2C12 cells.

Interestingly, we observed that only glucose deprivation significantly increased ARF1 expression (Fig. S1E and Fig. 1E, F). Moreover, glucose starvation not only promoted the expression of ARF1 mRNA but also upregulated genes such as ATP5F1, ATGL, and PLIN5 (Fig. 1G and Fig. S1G), indicating that glucose starvation enhances lipolysis and fatty acid oxidation. Consistent with many studies [10], glucose starvation also promoted AMPK phosphorylation (Fig. S1H, I), suggesting that energy-generating processes were activated.

To further confirm the contact between LDs and mitochondria, we isolated LDs and mitochondria from glucose-fed and glucose-starved C2C12 cells. We then examined the expression of the mitochondrial marker protein TOM20 in LDs and the LD marker protein Plin2 in mitochondria. First, we stained the extracted LDs using Bodipy (Fig. S1J). The results showed that the extracted LDs were morphologically intact. Western blot (WB) analysis demonstrated that the endoplasmic reticulum marker PDIA6 was undetectable in the isolated LDs (Fig. S1K)., confirming that the preparation was free from contamination by other organelles such as the endoplasmic reticulum and was therefore suitable for subsequent experiments. WB analysis revealed that GAPDH was undetectable in the isolated LDs (Fig. 1H), indicating that the LDs were not contaminated with cytoplasmic fractions. However, the TOM20 levels in the LDs from the glucose-starved group were significantly higher than those in the glucose-fed group (Fig. 1H). Similarly, GAPDH was undetectable in the isolated mitochondria (Fig. 1I), indicating no contamination of cytoplasmic fractions in the mitochondrial fraction. The mitochondria from the glucose-starved treatment group contained significantly more Plin2 than those from the glucose-fed group (Fig. 1I). These results suggest that glucose starvation facilitates the contact between LDs and mitochondria.

### Glucose starvation promotes the transfer of fatty acids from LDs to mitochondria

We next investigated whether glucose starvation induces the transfer of fatty acids stored in LDs to mitochondria. To examine the fate of fatty acids in LDs, we used a previously reported long-chain fatty acid (LCFA) tracking assay [22]. In this assay, C2C12 cells were pre-loaded with a fluorophore-labeled LCFA analog, Bodipy558/568-C12 (Red-C12). After pre-loading, cells were subjected to glucose starvation to limit nutrient uptake. Following the starvation period, LDs were labeled with Bodipy and mitochondria with MitoTracker. Under glucose-fed conditions, the Red-C12 signal was almost entirely localized within LDs (Fig. 1J). Interestingly, under glucose starvation, the Red-C12 signal appeared outside the LDs (Fig. 1J), indicating that fatty acids stored in the LDs were mobilized under these conditions. We then used Mander’s overlap coefficient to quantify the mobilization of LCFA in C2C12 cells. The overlap coefficient between Red-C12 and LDs was significantly lower in glucose-starved cells compared to glucose-fed cells (Fig. 1K). In glucose-fed conditions, there was minimal co-localization of Red-C12 with mitochondria (Fig. 1L). Importantly, glucose starvation resulted in strong co-localization of Red-C12 with mitochondria (Fig. 1L). The overlap coefficient of Red-C12 with mitochondria was greatly increased in glucose-starved cells compared to glucose-fed cells (Fig. 1M). These results suggest that glucose starvation induces the transfer of LCFAs from LDs to mitochondria.

### ARF1 regulates LD-mitochondrion contacts

**Fig. 2:**
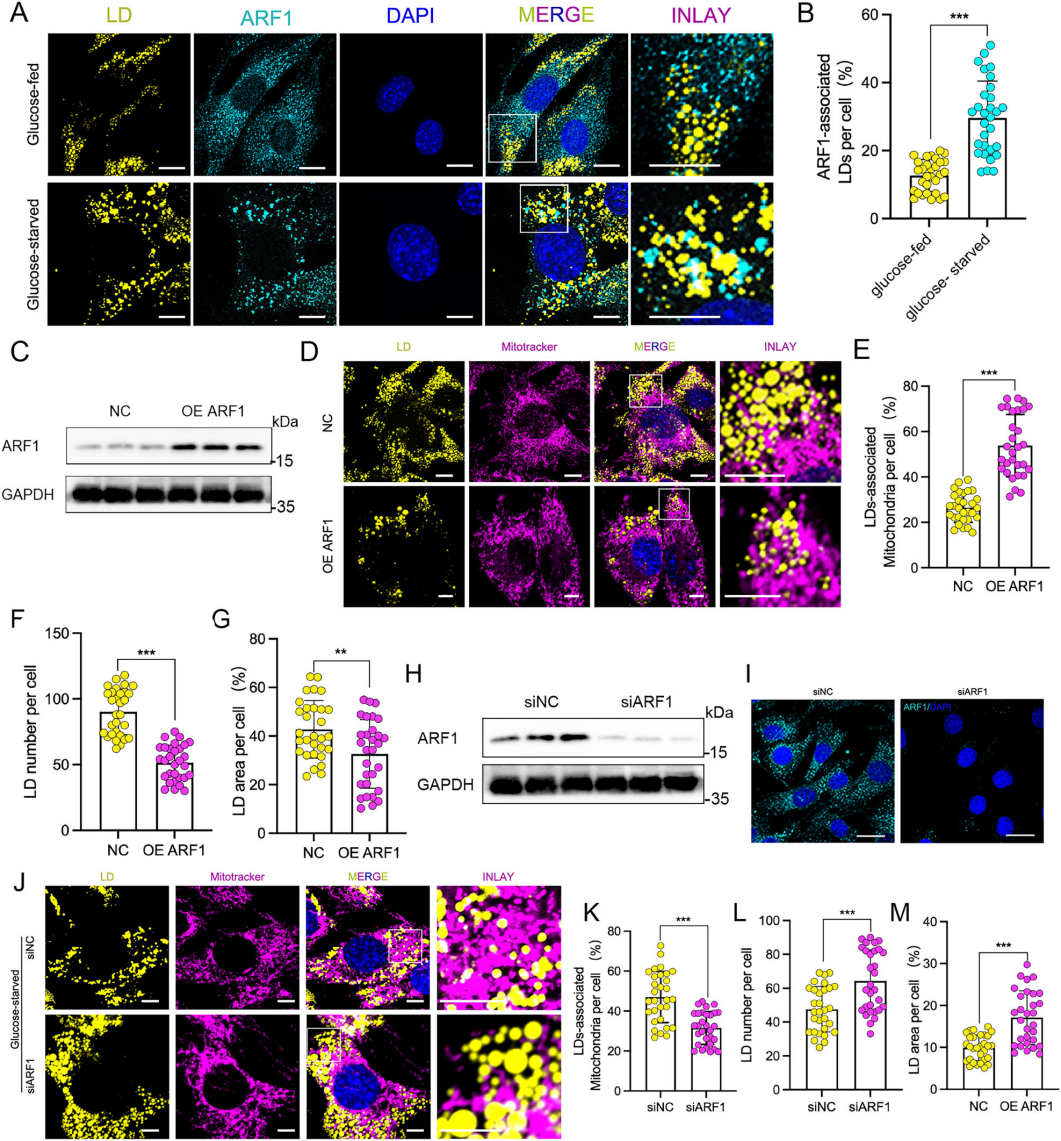
ARF1 regulates glucose starvation-induced LD-mitochondrion contacts. **A**, **B** Representative optical section images and quantification results showing ARF1 and LD contact in C2C12 cells immunostained with ARF1 antibody and LipidTOX (LD marker) under glucose-fed or glucose-starved conditions. Scale bar, 10 μm. **C** WB analysis of ARF1 in C2C12 cells transfected with ARF1 overexpression plasmid or empty vector (*n* = 3). **D**, **E** Representative optical section images and quantification results of mitochondrial and LD contacts in C2C12 cells transfected with ARF1 overexpression plasmid or empty vector (*n* = 3), immunostained using MitoTracker (mitochondrial marker) and BODIPY493/503 (LD marker). **F**, **G** Quantification of LD number and area per cell, *n* = 30 cells. **H**, **I** WB analysis and immunofluorescence staining of ARF1 in C2C12 cells transfected with ARF1 siRNA or empty vector (*n* = 3). **J**, **K** Representative optical section images and quantification results of mitochondrial and LD contacts in C2C12 cells transfected with ARF1 siRNA or empty vector (*n* = 3) and treated with glucose-fed or glucose-starved conditions. Cells were immunostained with MitoTracker and BODIPY493/503. Scale bar, 10 μm. **L**, **M** Quantification of the number (*n* = 30) and area (*n* = 30) of LDs per cell. **B**, **E**, **F**, **G**, **K–M** Data are presented as means ± SD of three biologically independent replicates, analyzed by two-sided unpaired Student’s t-test. *p < 0.05, **p < 0.01, ***p < 0.001.

Under conditions of glucose starvation, we observed a notable increase in the expression levels of ARF1 and sought to investigate its specific role during this metabolic stress. Previous studies have shown that ARF1 is localized on LDs (LDs) [23], prompting us to further explore the dynamic relationship between LDs and ARF1 during glucose starvation. Using an ARF1-specific antibody to label ARF1 and LipidTOX to label LDs in C2C12 cells subjected to glucose starvation, we found that starvation significantly enhanced the localization of ARF1 on LDs (Fig. 2A, B). Based on this, we hypothesized that ARF1 might regulate the interaction between LDs and mitochondria. To test this hypothesis, we overexpressed ARF1 in C2C12 cells to evaluate its effect on LD-mitochondrial contacts. We generated an ARF1 overexpression plasmid, transfected it into C2C12 cells, and confirmed the increased ARF1 protein levels by Western blotting (Fig. 2C). We also observed a marked increase in the co-localization of LDs and mitochondria in ARF1-overexpressing cells compared to controls (Fig. 2D). Mander overlap coefficient analysis revealed that ARF1 overexpression significantly enhanced LD-mitochondrial contact (Fig. 2E). Moreover, ARF1 overexpression led to a significant reduction in the number and area of LDs (Fig. 2F, G), suggesting that ARF1 promotes the depletion of LDs.

To further confirm the role of ARF1 in regulating LD-mitochondrial interactions, we used ARF1-targeting siRNA to knock down ARF1 expression in C2C12 cells. Western blotting and immunofluorescence analysis showed a significant reduction in ARF1 protein levels following siRNA transfection (Fig. 2H, I). After ARF1 knockdown, we subjected the cells to glucose starvation and examined the contact between LDs and mitochondria. We found that ARF1 knockdown significantly reduced the starvation-induced interaction between LDs and mitochondria (Fig. 2J, K), while concomitantly increasing the number and area of LDs (Fig. 2L, M). These results suggest that ARF1 plays a critical role in regulating LD-mitochondrial interactions and modulating LD dynamics during glucose starvation.

### ARF1 localizes at LD-mitochondrion contact sites and regulates fatty acid transfer

**Fig. 3:**
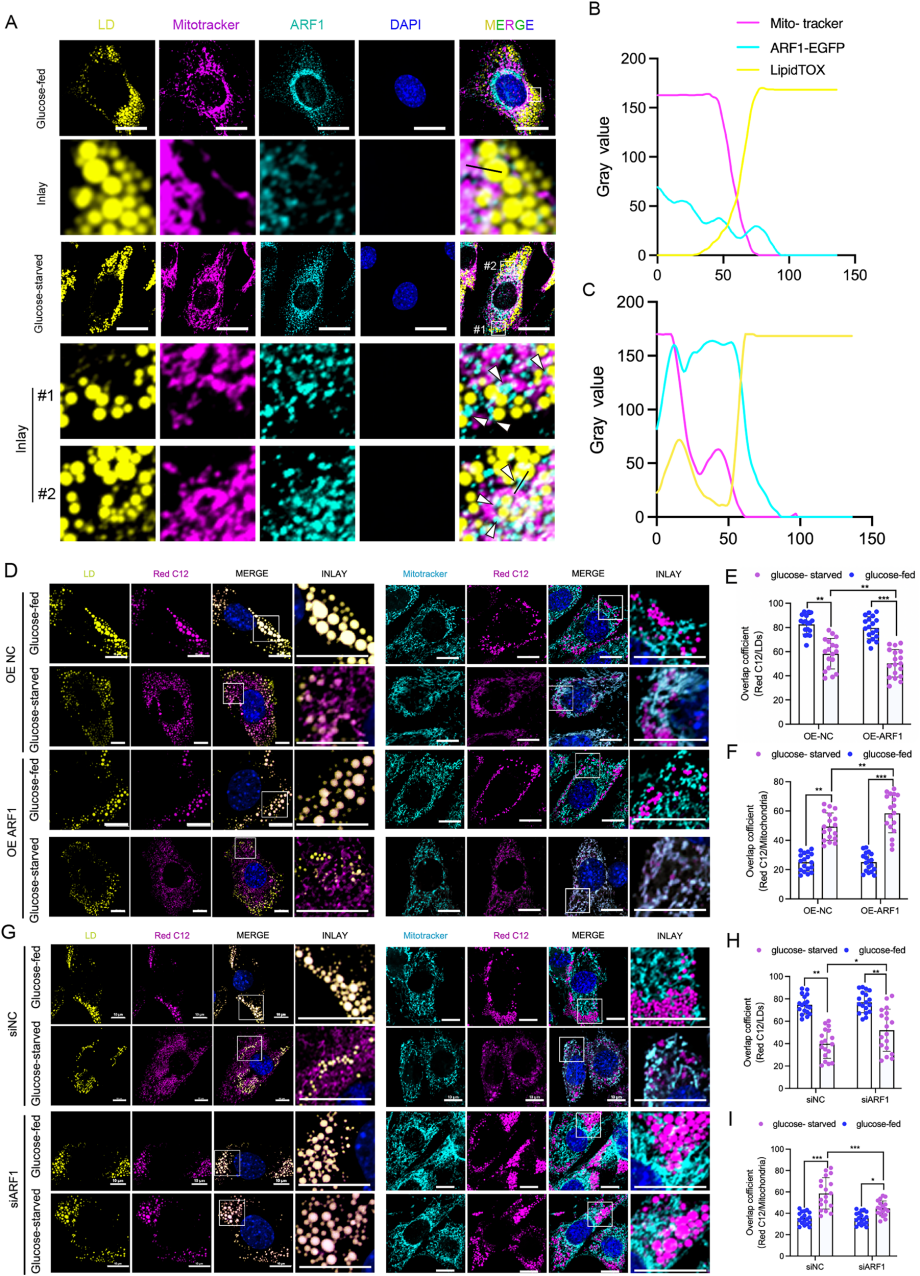
ARF1 localizes to LD-mitochondria contact sites and regulates fatty acid transfer. **A** Representative optical section images showing LD-mitochondrion contacts in C2C12 cells immunostained with MitoTracker (mitochondrial labeling, purple), LipidTOX (LD labeling, yellow), and ARF1-specific antibody. Cells were treated for 8 h under glucose-fed or glucose-starved conditions. Scale bar, 10 μm. (**B,** **C**) Fluorescence intensity profiles from the indicated line scans in panel A, showing the distribution of MitoTracker LipidTOX, and ARF1 labeling across the contacts between LDs and mitochondria. **D** Representative optical section images of C2C12 cells incubated with Red C12 for 16 h and then stained with MitoTracker (mitochondrial marker, green) and BODIPY493/503 (LD marker, green). Cells were transfected with ARF1 overexpression plasmid or empty vector and then treated for 8 h under glucose-fed or glucose-starved conditions. Scale bar, 2 μm. **E** Quantification of Red C12/LD overlap coefficient. *n* = 18 cells. **F** Quantification of Red C12/mitochondrial overlap coefficient. n = 18 cells. **G** Representative optical section images of C2C12 cells incubated with Red C12 for 16 hours and then stained with MitoTracker and BODIPY493/503. Cells were treated with Scrambled or ARF1 siRNA under glucose-fed or glucose-starved treatment conditions. Scale bar, 10 μm. **H** Quantification of Red C12/LD overlap coefficient. *n* = 24 cells. **I** Quantification of Red C12/mitochondrial overlap coefficient. *n* = 24 cells. **E**, **F**, **H**, **I** Data are presented as means ± SD of three biologically independent replicates, analyzed by two-way ANOVA followed by Turkey’s post-hoc test.

To investigate how ARF1 regulates the contact between LDs and mitochondria, we employed immunofluorescence staining to label ARF1, LDs, and mitochondria, allowing us to observe the spatial localization of ARF1 in relation to these organelles. Notably, our results revealed abundant ARF1 puncta at the contact sites between LDs and mitochondria under starvation conditions, whereas such localization was rarely observed in the control group (Fig. 3A–C). These findings strongly suggest that ARF1 directly regulates the contacts between LDs and mitochondria during starvation. To determine whether ARF1 is also involved in regulating fatty acid transfer between LDs and mitochondria, we examined the dynamics of fatty acids in cells overexpressing ARF1. Without ARF1 overexpression, Red-C12 signaling was predominantly enriched in LDs under glucose-fed conditions (Fig. 3D, E). Under glucose starvation, Red-C12 signaling significantly decreased in LDs and increased in mitochondria, indicating that starvation induced fatty acid transfer from LDs to mitochondria (Fig. 3D-F). Overexpression of ARF1 further reduced Red-C12 signaling in LDs and increased it in mitochondria after glucose starvation, suggesting that ARF1 overexpression facilitates the transfer of fatty acids from LDs to mitochondria (Fig. 3D-F).

Next, we used ARF1 siRNA to reduce ARF1 expression and observed fatty acid transport under glucose-starvation conditions. The results showed that reducing ARF1 expression under glucose starvation led to the accumulation of Red-C12 signaling in LDs and a reduction in mitochondria (Fig. 3G-I). This suggests that inhibiting ARF1 expression impairs the translocation of fatty acids from LDs to mitochondria. Taken together, these findings support the conclusion that ARF1 localizes to the contact site between LDs and mitochondria, where it regulates fatty acid transport from LDs to mitochondria during starvation conditions.

### GTP-Binding Active ARF1 Regulates LD-mitochondrion Interactions by Binding to Plin2

**Fig. 4:**
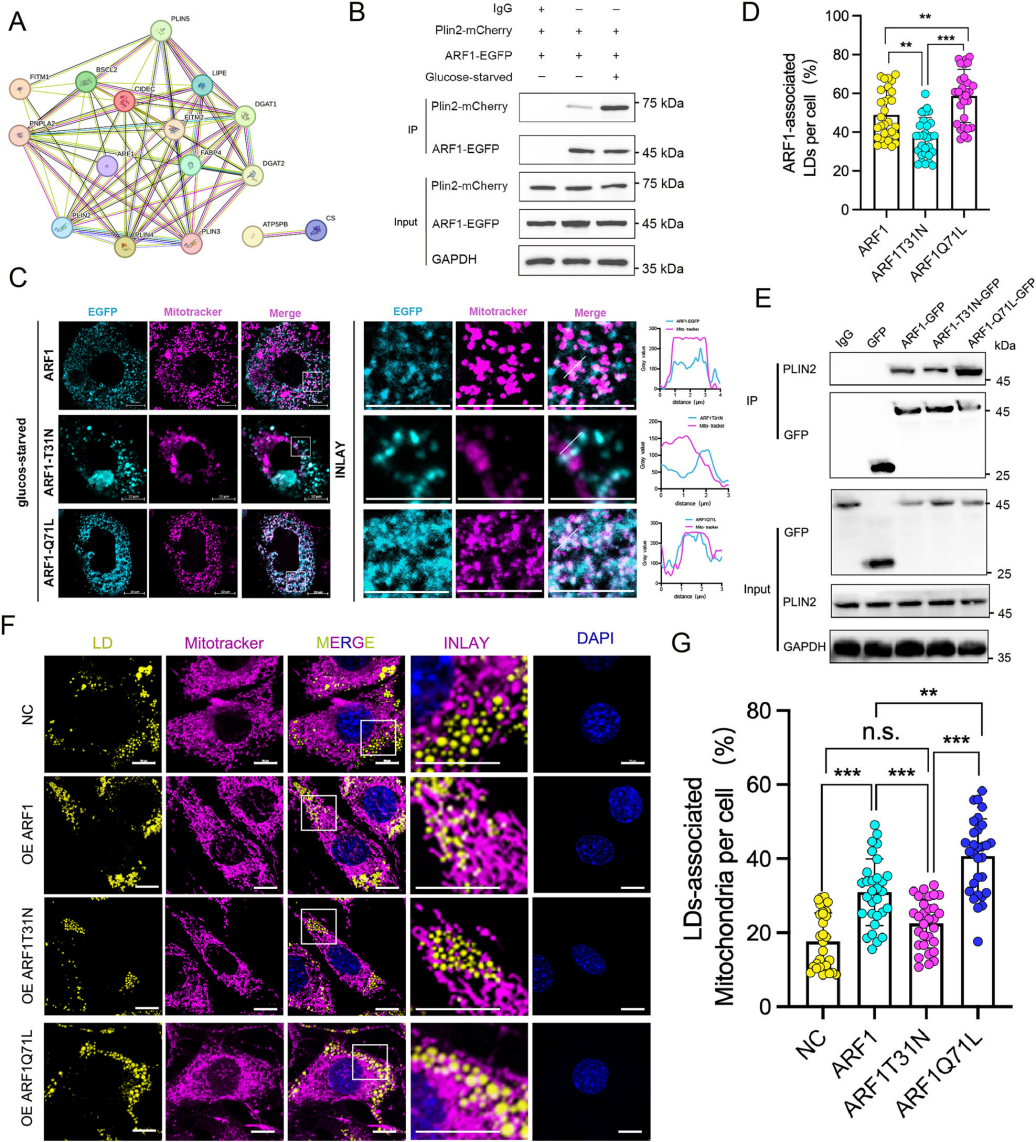
Active ARF1 mediates LD-mitochondrion contacts through interactions with Plin2. **A** Analysis of the interaction among ARF1 and proteins related to LDs. **B** Co-immunoprecipitation with an anti-EGFP antibody and western blotting conducted with the indicated antibodies. C2C12 cells transfected with Plin2-mCherry and ARF1-EGFP expression vectors were treated under glucose-fed or glucose-starved conditions for 8 h. **C**, **D** Representative optical section images and quantification results of ARF1-EGFP and mitochondrial contacts in C2C12 cells immunostained using MitoTracker (mitochondrial marker, red). Cells transfected with ARF1-EGFP, ARF1-T31N-EGFP, and ARF1-Q71L-EGFP plasmids were treated under glucose starvation conditions for 8 h. Scale bar, 10 μm. **E** Co-immunoprecipitation with an anti-EGFP antibody and western blotting conducted with the indicated antibodies. HEK293 cells were transfected with GFP, ARF1-EGFP, ARF1-T31N-EGFP, and ARF1-Q71L-EGFP expression vectors. **F**, **G** Representative optical section images and quantification results of LD-mitochondrion contacts in C2C12 cells immunostained using MitoTracker (mitochondrial marker, red) and BODIPY493/503 (LD marker, green). Cells transfected with ARF1-EGFP, ARF1-T31N-EGFP, and ARF1-Q71L-EGFP plasmids were treated under glucose starvation conditions for 8 h. Scale bar, 10 μm. **D**, **G** Data are presented as means ± SD of three biologically independent replicates, analyzed by one-way ANOVA followed by Turkey’s post-hoc test. *p < 0.05, **p < 0.01, ***p < 0.001.

To investigate whether ARF1-regulated mitochondrial interactions require the involvement of other proteins, we used protein interaction prediction analysis and found that ARF1 interacts with Plin2 (Fig. 4A). We co-transfected ARF1-GFP and Plin2-mCherry into 293 T cells and examined their interactions after glucose starvation. The results showed that glucose starvation led to an increase in ARF1-GFP and Plin2-mCherry interactions compared to the glucose-fed group, which was consistent with the glucose starvation-induced increase in LD-mitochondrion interactions (Fig. 4B). These interactions suggest that ARF1 may act as a mitochondrial junction protein. To determine whether ARF1 localizes to mitochondria under starvation, we used immunofluorescence staining to observe the positional relationship between ARF1 and mitochondria. We found that glucose starvation significantly increased the co-localization of ARF1 with mitochondria (Fig. 4C).

We next examined whether ARF1 activity affects its mitochondrial localization. We expressed WT ARF1-EGFP, a GTP-bound active ARF1-Q71L-EGFP mutant, and a GDP-bound inactive ARF1-T31N-EGFP mutant in cells and examined their localization to mitochondria under glucose starvation. The results showed that both ARF1-EGFP and ARF1-Q71L-EGFP strongly co-localized with mitochondria under glucose starvation conditions, with ARF1-Q71L-EGFP having a higher degree of overlap with mitochondria compared to ARF1-EGFP. In contrast, ARF1-T31N-EGFP had significantly lower co-localization with mitochondria (Fig. 4B, D). These results suggest that glucose starvation promotes the mitochondrial localization of active ARF1, which may allow ARF1 to bind more Plin2 and regulate LD-mitochondrion interactions.

To test this hypothesis, we transfected wild-type ARF1-GFP and mutated ARF1 plasmids into 293 T cells and detected their binding to endogenous Plin2 using immunoprecipitation (IP). The results showed that wild-type ARF1 and ARF1-Q71L had higher binding abilities to Plin2 compared to ARF1-T31N (Fig. 4E). Additionally, we overexpressed wild-type ARF1-GFP and mutated ARF1 plasmids in C2C12 cells and examined LD-mitochondrion interactions. Overexpression of wild-type ARF1 and ARF1-Q71L significantly increased the contact between LDs and mitochondria compared to the control group, with ARF1-Q71L showing a higher interaction level than wild-type ARF1 (Fig. 4F, G). Overexpression of ARF1-T31N did not significantly increase the contact between LDs and mitochondria (Fig. 4F, G). These results indicate that GTP-binding active ARF1 regulates LD-mitochondrion interactions by binding to Plin2, particularly under glucose starvation conditions.

### AMPK Regulates ARF1-Mediated LD-mitochondrion Interactions

We sought to understand the signaling mechanisms that regulate ARF1-mediated LD-mitochondrion contacts. Given that AMPK is a central regulator of starvation responses and energy metabolism [24], we hypothesized that AMPK might be involved in regulating these interactions under glucose starvation conditions.

**Fig. 5:**
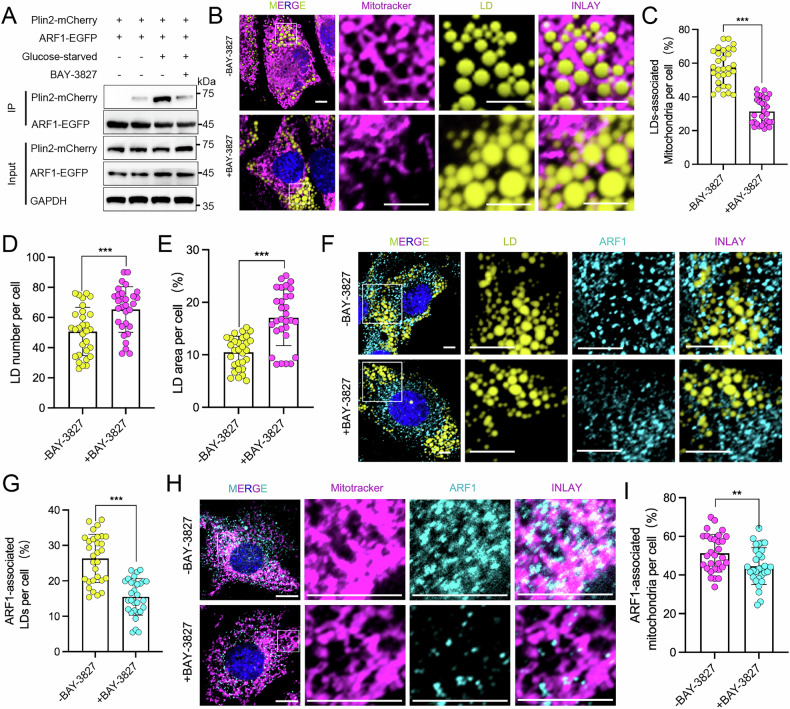
AMPK regulates ARF1-mediated contact of LDs with mitochondria. **A** Immunoprecipitation using an anti-EGFP antibody and immunoblotting with the same antibody. Cells transfected with Plin2-mCherry and ARF1-EGFP expression vectors were treated or not with BAY-3827 (5 μM, 8 h) under glucose-fed or glucose-starved conditions (*n* = 3). **B**, **C** Representative optical section images and quantitative analysis of LD-mitochondrion contacts in C2C12 cells immunostained with MitoTracker (mitochondrial marker, purple) and LipidTOX (LD marker, yellow). Cells were treated with or without BAY-3827 for 8 hours under glucose starvation conditions (*n* = 30). Scale bar, 10 μm. **D**, **E** Quantification of the number (*n* = 30) and area (*n* = 30) of LDs per cell. **F**, **G** Representative optical section images and quantification results of ARF1 and LD contacts in C2C12 cells immunostained with ARF1 antibody (green) and LipidTOX (LD marker, green). Cells were treated with or without BAY-3827 for 8 hours under glucose starvation conditions (*n* = 30). Scale bar, 10 μm. **H**, **I** Representative optical section images and quantification results of ARF1 and mitochondrial contacts in C2C12 cells immunostained with ARF1 antibody (light blue-green) and MitoTracker (mitochondrial marker, purple). Cells were treated with or without BAY-3827 for 8 h under glucose starvation conditions (*n* = 30). Scale bar, 10 μm. **C**–**E**, **G**, **I** Data are presented as means ± SD of three biologically independent replicates, analyzed by two-sided unpaired Student’s t-test. *p < 0.05, **p < 0.01, ***p < 0.001.

To test this hypothesis, we co-transfected ARF1-EGFP and Plin2-mCherry into C2C12 cells, subjected the cells to glucose starvation, and treated them with or without the AMPK inhibitor BAY-3827. We then examined the binding between ARF1-EGFP and Plin2-mCherry by immunoprecipitation. The results showed that the binding of ARF1-EGFP to Plin2-mCherry was significantly enhanced under glucose starvation, whereas the addition of BAY-3827 significantly reduced this binding (Fig. 5A). Additionally, BAY-3827 treatment significantly diminished the starvation-induced interaction between LDs and mitochondria (Fig. 5B, C), and led to an increase in both the number and area of LDs (Fig. 5D, E).

Next, we assessed the effect of BAY-3827 on the dynamics of ARF1 localization in LDs and mitochondria using fluorescence imaging. We found that AMPK inhibition by BAY-3827 resulted in a significant reduction in ARF1 localization on LDs and mitochondria compared to controls (Fig. 5F-I).

Taken together, these findings suggest that AMPK regulates ARF1-mediated LDs-mitochondria contact, facilitating lipid mobilization in response to energy demand changes by modulating the subcellular localization of ARF1.

### Inhibition of ARF1 Leads to Lipid Accumulation in Skeletal Muscle of Mice Under Starvation Conditions

**Fig. 6:**
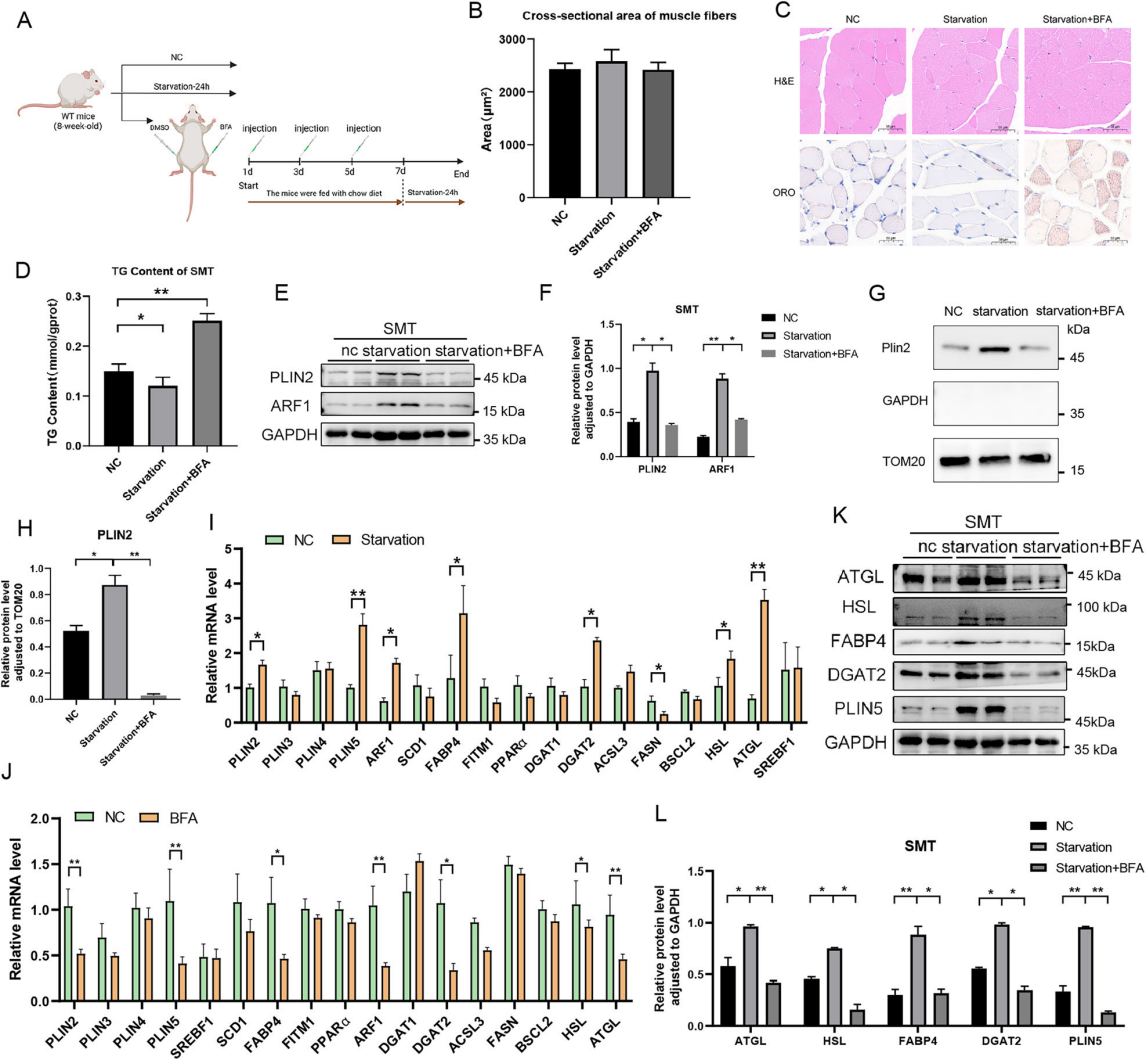
Inhibition of ARF1 Activity Leads to Starvation-Induced Reduction of LD-mitochondrion Interactions and Lipid Accumulation. **A** Schematic representation of mice starved with or without BFA injection treatment. **B** Comparative statistical analysis of muscle cross-sectional area in mice. **C** HE staining and Oil Red O staining of mouse muscle tissue sections. **D** Determination of triglyceride content in mouse muscle tissue. **E** WB detection of PLIN2 and ARF1 protein expression levels in muscle tissues of mice after starvation and BFA treatment. **F** Quantification of WB results for ARF1 and PLIN2, normalized to GAPDH in group E. **G** WB detection of PLIN2, GAPDH, and TOM20 protein expression levels in muscle tissue mitochondria after starvation and BFA treatment. **H** Quantification of WB results for PLIN2, normalized to TOM20 in group G. **I** Detection of mRNA expression levels of lipid metabolism-related genes in muscle tissues using qPCR after starvation or feeding treatment in mice. **J** Detection of mRNA expression levels of lipid metabolism-related genes in muscle tissues using qPCR after mice were treated with or without BFA under starvation conditions. **K** WB analysis of lipid metabolism-related proteins in skeletal muscle tissues from nc, starvation, and starvation+BFA groups. GAPDH was used as a loading control. **L** Quantification of protein levels normalized to GAPDH. **B**, **D**, **F**, **H**, **I**, **J**, **L** Data are presented as means ± SD of three biologically independent replicates, analyzed by one-way ANOVA followed by Turkey’s post-hoc test.*p < 0.05, **p < 0.01, ***p < 0.001.

To investigate the effect of ARF1 in vivo on regulating LD-mitochondrion interactions, we established a mouse starvation model as well as a mouse starvation and injection of ARF1 inhibitor BFA model (Fig. 6A). At the end of starvation, mouse muscle tissue was collected and the muscle fiber cross-sectional area was examined. The results showed that the cross-sectional area of myofibers did not exhibit significant differences between different experimental groups (Fig. 6B), indicating that the size and morphological structure of mouse gastrocnemius muscle fibers remained relatively stable under starvation conditions.

Mouse muscle tissues were stained with HE and oil red O to examine their histomorphology and fat accumulation. The oil red O staining results showed a decrease in oil red positive staining in the starvation-treated group, but a significant increase in positive staining was observed in the muscles of mice treated with BFA (Fig. 6C). Consistent with this, starvation resulted in a significant decrease in muscle tissue triglycerides, while BFA treatment led to a significant increase in triglycerides (Fig. 6D), suggesting that inhibition of ARF1 results in lipid accumulation in muscle tissue. Interestingly, starvation treatment significantly promoted the expression levels of ARF1 and Plin2 proteins in mouse muscle (Fig. 6E, F), whereas BFA treatment significantly decreased ARF1 and Plin2 protein levels (Fig. 6E, F), again suggesting that ARF1 may be critical in response to starvation.

We next isolated mitochondria from muscle tissues to test whether starvation induces contact between LDs and mitochondria in mouse muscle tissues. Western blot assay results showed the absence of GAPDH in the extracted mitochondria (Fig. 6G), suggesting that there was no contamination of cytoplasmic fractions in the isolated mitochondria. Interestingly, starvation treatment resulted in a significant increase in the content of Plin2 in mitochondria (Fig. 6G, H), suggesting that starvation treatment promotes LD-mitochondrion interactions in muscle tissue. However, the presence of Plin2 was almost undetectable in the mitochondria of BFA-injected mice after starvation treatment (Fig. 6G, H), indicating that inhibition of ARF1 blocks LD-mitochondrion interactions.

To further investigate the effects of starvation and BFA treatment on lipid metabolism in mouse gastrocnemius muscle, we examined the expression of genes related to lipid metabolism in each group. The findings showed that starvation treatment promoted the expression of ARF1 in the muscle group (Fig. 6I), consistent with observations in cells. In addition, starvation upregulated the mRNA and protein expression of Plin2, Plin5, FATP4, DGAT2, ATGL, and HSL (Fig. 6I, K, L), which were downregulated after starvation in BFA-injected mice (Fig. 6J-L), suggesting that inhibition of ARF1 suppresses lipid mobilization.

## Discussion

Under starvation conditions, mitochondrial β-oxidation is essential for generating energy to maintain normal cellular function [23]. It has been shown that contact between LDs and mitochondria promotes mitochondrial β-oxidation [11]. In this study, we demonstrate that under starvation conditions, AMPK facilitates the localization of active ARF1 to mitochondria, enhancing the binding between ARF1 and Plin2. This interaction ultimately increases the contact between LDs and mitochondria, regulating fatty acid transfer and promoting mitochondrial β-oxidation. In vivo experiments revealed that starvation induces an increase in LD-mitochondrion contacts and upregulates ARF1 expression in mouse muscle tissues. Conversely, administration of ARF1 inhibitors to mice muscle blocked the contact between LDs and mitochondria, resulting in the downregulation of genes related to β-oxidation.

ARF1 is essential for the regulation of membrane transport, lipid metabolism, and organelle function in cells. Previous studies have highlighted ARF1’s critical role in the transport of ATGL and PLIN1 to the surface of LDs [25]. When ARF1 binds to GTP, it can act directly on the surface of LDs through the COPI mechanism, mediating the emergence and sprouting of LDs with a diameter of 60–100 nm from the LDs [26]. This mechanism is essential for regulating the surface properties of LDs at different metabolic stages within the cell and facilitates the link between LDs and the endoplasmic reticulum [16]. Consistent with our present study, recent research has also shown that ARF1 is present at contact sites between LDs and mitochondria, LDs and peroxisomes, and among the three organelles. ARF1 in the GTP-bound state increases contacts between LDs, mitochondria, and the endoplasmic reticulum [20]. These results suggest that ARF1 is a key factor in regulating lipid metabolism, mediating lipid transport, storage, and oxidation in multiple ways.

LD-mitochondrion interactions occur in a wide variety of cell types [12, 27–29], yet their functions and significance remain not fully understood. Current evidence suggests that these interactions have two distinct roles depending on the cell type or metabolic state. Firstly, in cells such as skeletal muscle and hepatocellular carcinoma cell [10, 11, 30], the contact between LDs and mitochondria facilitates the transfer of fatty acids from LDs to mitochondria, promoting energy expenditure. This process is essential for generating ATP through mitochondrial β-oxidation, especially under energy-demanding conditions such as starvation, where it helps mitigate lipotoxicity by efficiently mobilizing stored lipids [31]. Secondly, in adipocytes [32] and liver [33], the contact between LDs and mitochondria promotes the accumulation of lipids within LDs to reduce energy consumption. During adipocyte differentiation, mitochondria provide ATP for lipid synthesis, aiding in the expansion of LDs [34, 35]. Thus, the interaction between these organelles is dynamic and context-dependent, with different functions tailored to the cell’s metabolic needs.

Under energy-demanding conditions, the contact between LDs and mitochondria enhances the transfer of fatty acids, which is thought to help mitigate lipotoxicity [36]. Although LD-mitochondrion interactions have been extensively studied, there is still much to learn about their tethering and regulatory mechanisms. In myoblasts, Plin5 can independently mediate LD-mitochondrion interactions, as its C-terminus contains a region that targets mitochondria [37, 38]. Interestingly, Rab8a [10] and FATP4 [11] can each act as receptors for Plin5 on mitochondria, thereby linking LDs to mitochondria. Additionally, Plin2 mediates LD-mitochondrion interactions under various conditions. For instance, starvation induces interactions between Plin2 and CPT1A [30], as well as with ARF1 (as shown in this study). In contrast, Aflatoxin B1 promotes Plin2 binding to p53 [39], mediating LD-mitochondrion interactions. In brown adipose tissue, Plin1 and MFN2 are involved in these interactions [40]. These findings indicate that perilipin family proteins play crucial roles in mediating LD-mitochondrion interactions, and highlight the existence of multiple mitochondrial junction proteins for the same perilipin protein. This reflects the complex and dynamically changing regulatory mechanisms underlying LD-mitochondrion interactions.

Under serum starvation conditions, AMPK phosphorylates Rab8a, promoting its binding to Plin5 to regulate LD-mitochondrion interactions [10]. Our results indicate that glucose starvation enhances AMPK phosphorylation, while AMPK activation facilitates ARF1 translocation to mitochondria, suggesting that the mitochondrial localization of ARF1 is crucial for regulating LD-mitochondrion interactions. Inhibition of AMPK activity significantly reduced the localization of ARF1 to mitochondria and decreased the contact between LDs and mitochondria. Furthermore, under energy-deprived conditions, AMPK activates and remodels the de-tyrosinated microtubule network, increasing LD mobility to facilitate organelle redistribution and enhance LD-mitochondrion contacts [41, 42]. These findings exemplify the diverse roles of AMPK in regulating LD-mitochondrion interactions. Additionally, recent evidence in hepatocellular carcinoma cells suggests that glucose starvation promotes the phosphorylation of PFKL, which facilitates its binding to Plin2. This interaction subsequently leads to the phosphorylation of Plin2, enhancing its binding to CPT1A and ultimately facilitating LD-mitochondrion interactions and β-oxidation in mitochondria [30]. However, whether PFKL-regulated phosphorylation of Plin2 can influence the interactions between Plin2 and ARF1 remains to be further investigated. Considering that ARF1 and CPT1A are distinct proteins in both function and structure, we hypothesize that the regulatory mechanisms governing the interactions between Plin2 and either ARF1 or CPT1A may differ. At the very least, the results of the present study suggest that the binding of Plin2 to ARF1 is regulated by AMPK, although the specific regulatory mechanisms need to be further explored, including how AMPK regulates the translocation of ARF1 to mitochondria.

Overall, our study identifies a novel mechanism by which ARF1, regulated by AMPK, enhances LD-mitochondrion interactions to facilitate fatty acid transfer and promote mitochondrial β-oxidation under starvation conditions. This mechanism is essential for maintaining energy homeostasis and cellular function during nutrient deprivation. Further research could explore potential therapeutic targets within this pathway for treating metabolic diseases characterized by impaired lipid metabolism.

## Supplementary information

Supplementary materials -western blot source data

Supplementary Figure 1

Supplementary materials-qPCR source data

## Data Availability

The data used to support the findings of this study are available from the corresponding author upon request.
