# Supplementary figures and images for: AMPK regulates ARF1 localization to membrane contact sites to facilitate fatty acid transfer between lipid droplets and mitochondria
Source: Cell Death Dis. 2025 Aug 18;16(1):623. doi: 10.1038/s41419-025-07957-7 (PMC12361384; doi:10.1038/s41419-025-07957-7)

Source data

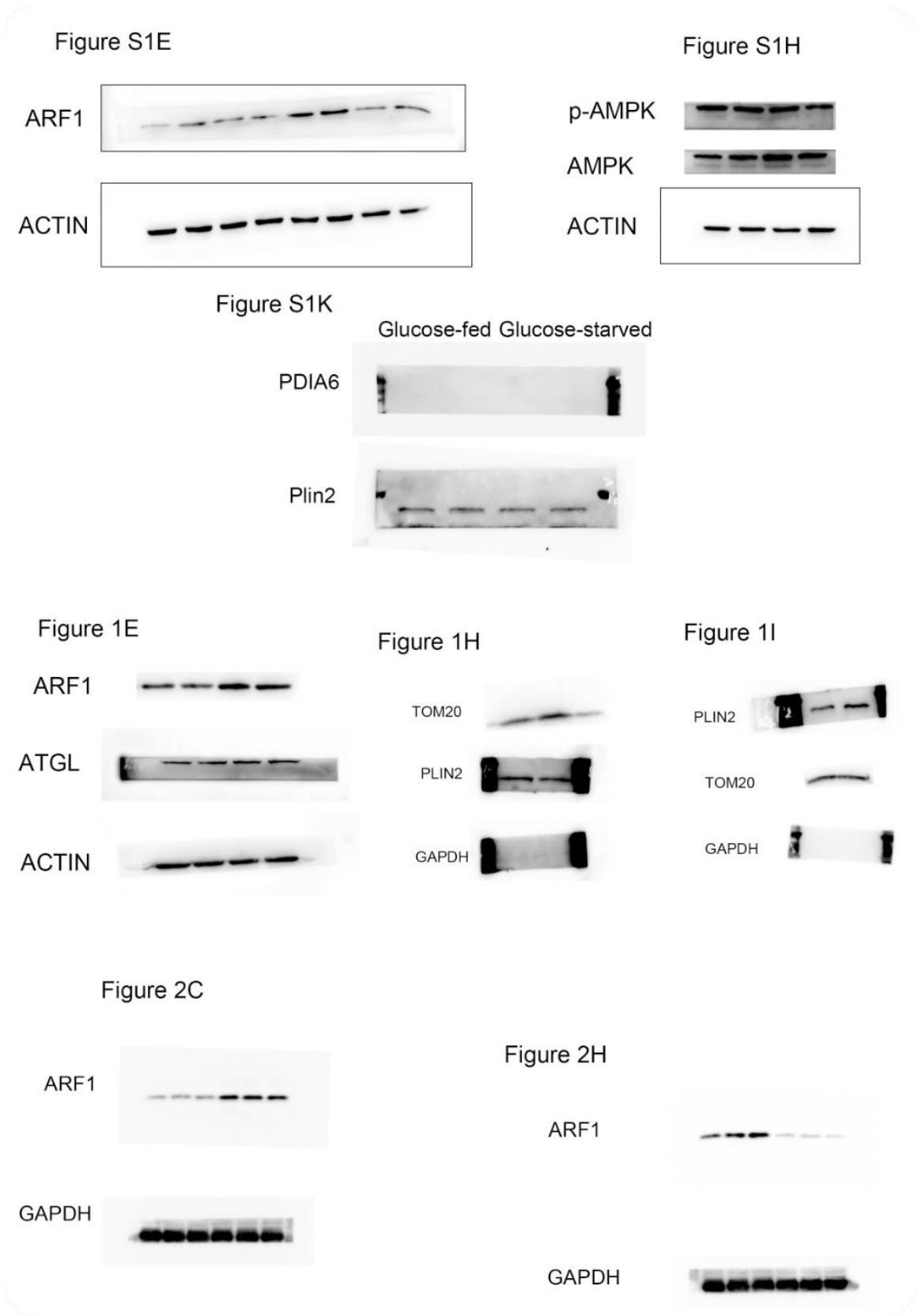

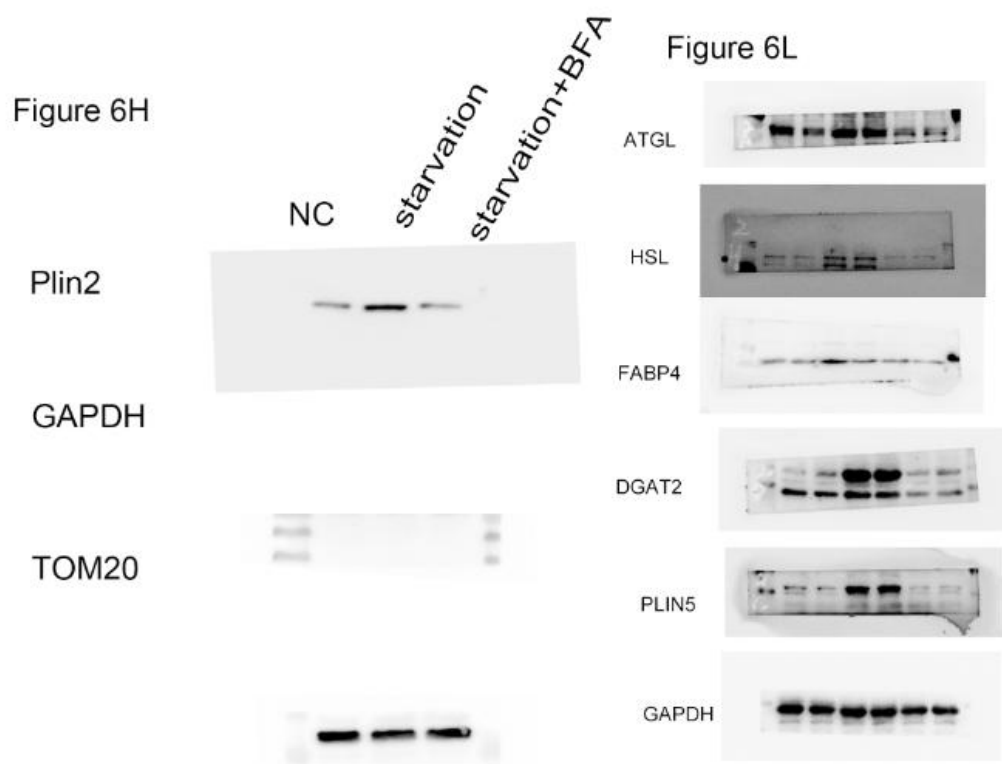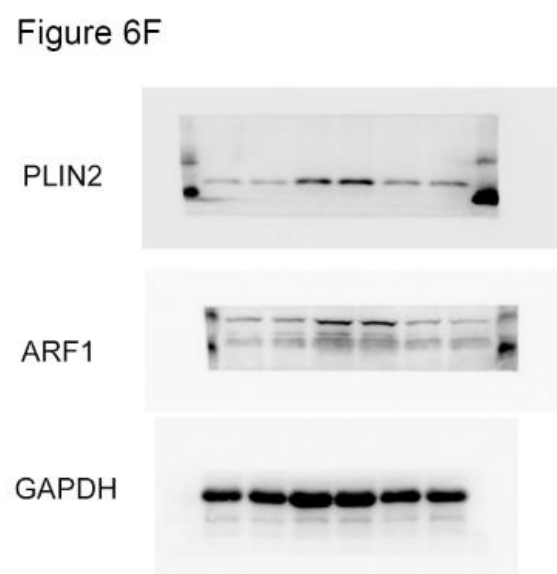

Figure 4B

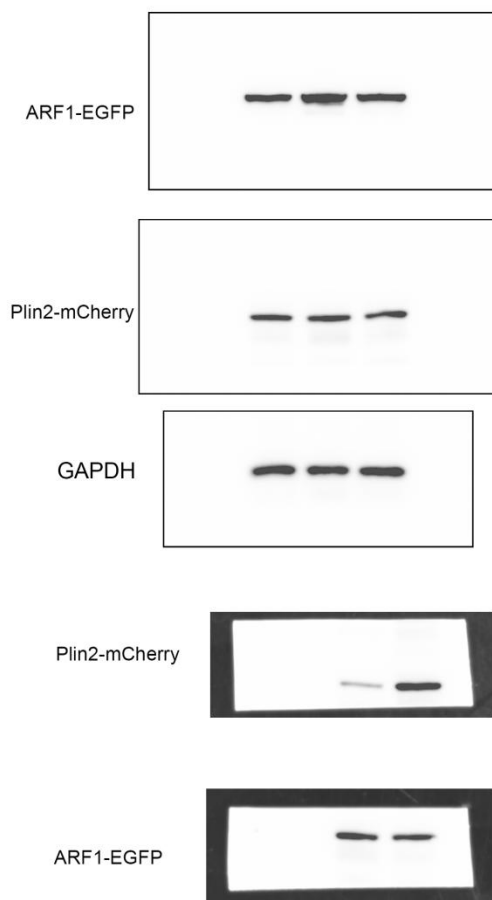

Figure 4E

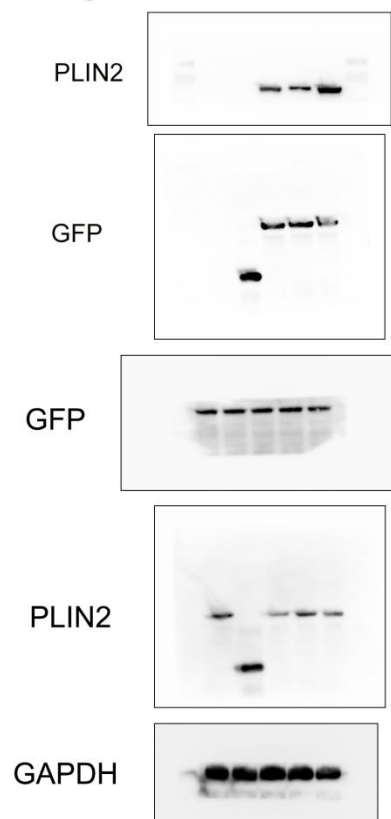

Figure 5A

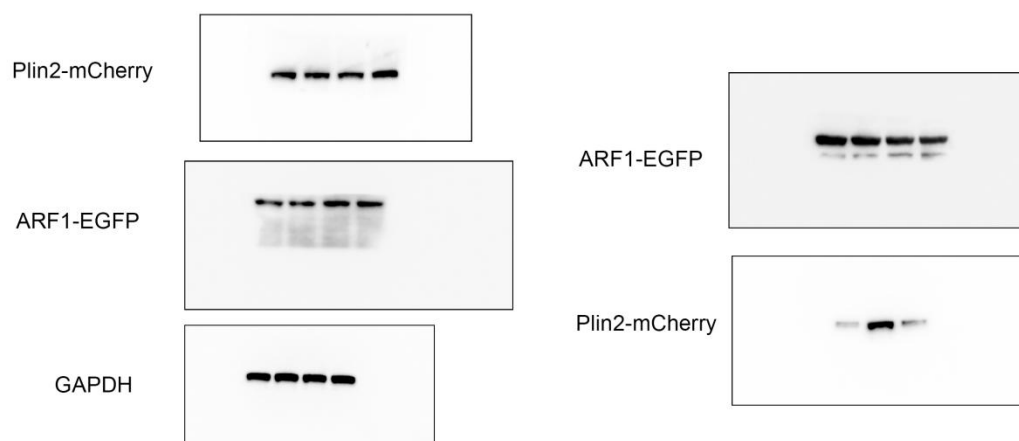

Supplement: Supplementary file 1 — Supplementary materials -western blot source data [file 41419_2025_7957_MOESM1_ESM.pdf]
