# Supplementary material for: AMPK regulates ARF1 localization to membrane contact sites to facilitate fatty acid transfer between lipid droplets and mitochondria
Source: Cell Death Dis. 2025 Aug 18;16(1):623. doi: 10.1038/s41419-025-07957-7 (PMC12361384; doi:10.1038/s41419-025-07957-7)
Supplement: Supplementary file 2 — Supplementary Figure 1 [file 41419_2025_7957_MOESM2_ESM.pdf]

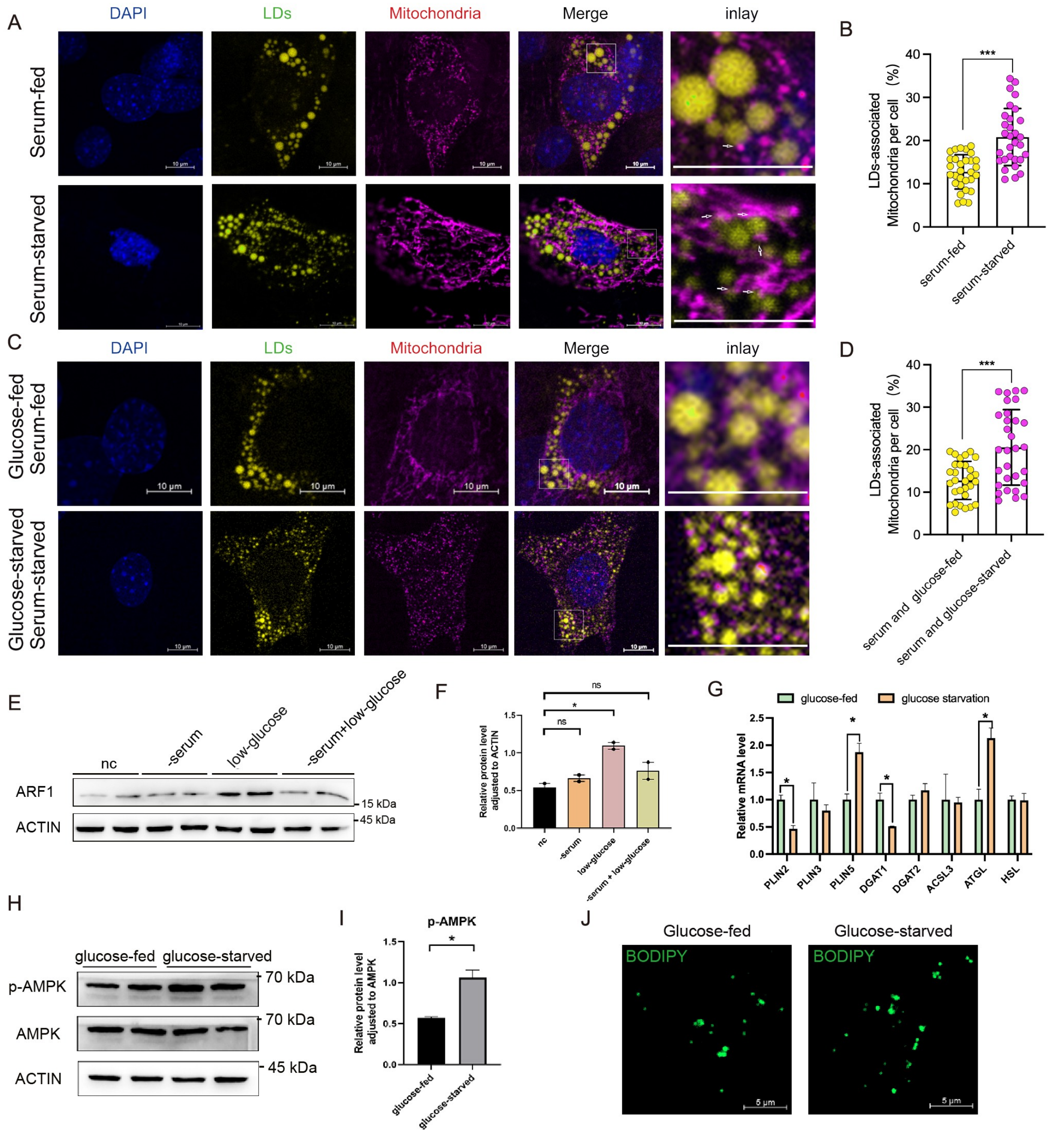

Supplementary Fig. 1 Glucose starvation promotes LD-mitochondrion interactions and ARF1 expression.

(A-D) Representative optical section images and quantification results of LD-mitochondrion contacts after staining of C2C12 cells treated for 8 hr with serum starvation (A and B) or glucose and serum starvation (C and D) using MitoTracker (mitochondrial marker, red) and BODIPY493/503 (LD marker, green). Scale bar, 10 μm.

(E) Western blot (WB) analysis of ARF1 in C2C12 cells from various starvation-treated groups (n = 3) and controls (n = 3).

(F) Quantification of WB results of ARF1, normalized to ACTIN in panel E.

(G) Expression of lipid metabolism-related genes after glucose starvation treatment.

(H) AMPK and p-AMPK level after glucose starvation.

(I) Quantification of WB results of p-AMPK, normalized to ACTIN in panel H.

(J) Representative optical section images after staining of isolated lipid droplets using BODIPY.* $p < 0.05$, ** $p<0.01$.

(K) WB analysis of PDIA6 and PLIN2 was performed on lipid droplets isolated from C2C12 cells subjected to various starvation treatments (n = 3) and controls (n = 3).
